# A murine coronavirus infection platform identifies proviral and proinflammatory activities of SARS-CoV-2 accessory protein 7a

**DOI:** 10.1128/jvi.01961-24

**Published:** 2025-12-16

**Authors:** Grant M. Hawkins, Enya Qing, Julisa Salgado, Pearl Chan, Edward M. Campbell, Stanley Perlman, Tom Gallagher

**Affiliations:** 1 Department of Microbiology and Immunology, Loyola University Chicago, Maywood, Illinois, USA; 2 Department of Microbiology and Immunology, University of Iowa 311821, Iowa City, Iowa, USA; University of North Carolina at Chapel Hill, Chapel Hill, North Carolina, USA

**Keywords:** coronavirus, SARS-CoV-2, accessory proteins, interferon, virus replication, macrophage, proinflammatory cytokines

## Abstract

Severe acute respiratory syndrome coronavirus 2 (SARS-CoV-2) and related sarbecoviruses encode a set of accessory proteins (3a, 3b, 6, 7a, 7b, 8, 9b, and 10) that control host responses to infection and promote virus growth. Of these accessory proteins, 7a is set apart by its intracellular localization near CoV budding sites and its incorporation into secreted virions. To investigate 7a functions during CoV infections under biosafety level 2 conditions, we constructed recombinant mouse hepatitis viruses (rMHVs) (rMHV strain A59) that express sarbecovirus 7a genes. Comparative infections revealed that 7a increased viral replication and viral output in immortalized murine cell cultures and in primary bone marrow-derived macrophages (BMDMs). This proviral effect was independent of a previously reported 7a-mediated interferon antagonizing activity. 7a is a type I transmembrane protein with a short cytoplasmic tail that operates in subcellular trafficking and signal transduction. To further elucidate tail functions, we generated a set of rA59 viruses expressing substitutions in the tail di-lysine motifs. Several substitutions reduced 7a proviral activities; notably, the K119A change in rA59-7a-KRATE eliminated 7a support of virus yield. The K119A change also reduced mouse-adapted SARS-CoV-2 virus yields in infected BALB/c mice. 7a expression was proinflammatory in BMDMs, as measured by cytokine arrays. Cytoplasmic tail substitutions tempered these proinflammatory responses, implying connections with proviral activities. SARS-CoV-2-infected macrophages have been implicated in inflammatory COVID-19, and these findings point to 7a cytoplasmic tails as potential contributors to cytokine-mediated disease.

**IMPORTANCE:**

This study shows that SARS-CoV-2 accessory protein 7a promotes infection of a phylogenetically distinct embecovirus and, in doing so, elicits proinflammatory and potentially disease-relevant host responses. The proviral and proinflammatory activities were traced in part to a short 7a cytoplasmic tail. The findings localize and highlight a specific proviral component in a sarbecovirus accessory protein.

## INTRODUCTION

Enveloped, positive-sense RNA-containing coronaviruses encode non-structural proteins (nsps 1–16) necessary for viral RNA-dependent RNA replication and transcription, virion structural proteins (spike [S], envelope [E], membrane [M], and nucleocapsid [N]), and several accessory proteins. These accessory proteins vary between coronavirus genera; together they form a diverse collection of host-modulating factors (1–3). By definition, the accessory proteins are non-essential in many *in vitro* infection environments, yet they contribute significantly to *in vivo* virus virulence by suppressing antiviral immune responses and by supporting virus production (4–9).

Severe acute respiratory syndrome coronavirus 2 (SARS-CoV-2) encodes accessory proteins 3a, 3b, 6, 7a, 7b, 8, 9b, and 10 (1, 2, 10–12). Deleting one or more of these genes frequently attenuates *in vivo* virus virulence (4–7, 13). Several accessory gene products, notably 3a and 6, have clear proviral functions that are also evident *in vitro* (4, 14, 15). Here we became drawn to protein 7a, in large part because of its particular structural features and wide range of reported functions. 7a is a virion-associated type I transmembrane protein (16). The 87-amino-acid Ig-like 7a ectodomain may bind alternative host cell-surface proteins and hypothetically expand virus spread (17–19). 7a accumulation within cells also generates several potentially proviral and proinflammatory outcomes by restricting intracellular transport and function of MHC-I (20), SERINC5 (21), and tetherin (22); by interfering with autophagy (23–25); or by inducing apoptosis (26), endoplasmic reticulum (ER) stress (26), and inflammation (27, 28).

Some of these varied responses may trace to a short five-amino-acid 7a cytoplasmic tail (29). The carboxy-terminal tail encodes a canonical ER retention motif, which potentially interacts with COPI coatomer complexes, possibly holding 7a in position for incorporation into virions (16). Intracellular retention traps both 7a and 7a-associated MHC-I molecules in the ER, reducing viral antigen presentation (20). While embedded in intracellular membranes, 7a tails bind antiapoptotic BclXL (26), affecting apoptotic responses. 7a tails interact with RNF121, an E3 ubiquitin ligase (27) which generates a membrane-associated polyubiquitin substrate that then accumulates secondary effectors that block STAT2 phosphorylation (30, 31) and activate NF-kB, ultimately modulating proinflammatory responses (27, 28, 32).

Of note, nearly all of these reported mechanisms by which 7a effects functional responses have come from experiments in which the 7a proteins were overexpressed in the absence of infection and/or modified by epitope tags at carboxy-terminal tails. These experimental approaches have value but may not reflect unaltered 7a biology in the natural infection contexts that include many diverse virus–host interactions. Therefore, we aimed to evaluate 7a mechanisms operating during a complete infection process, using an orthologous murine coronavirus that can be investigated in biosafety level 2 laboratories. Rodent-specific embecoviruses have been proven to be useful tools for assessing functions of human pathogenic sarbecovirus accessory proteins (33, 34). The embecovirus subgenus does not encode any accessory proteins homologous to SARS-CoV-2 (35–37), making it so that 7a might singularly add unique functions to embecovirus infections. For these reasons, we constructed recombinant mouse hepatitis embecoviruses encoding SARS-CoV-2 7a (rA59-7a) and then evaluated 7a-specific functions. The tests were facilitated by prior knowledge that antiviral host responses to MHV infection (38, 39) can be measured *ex vivo* in immunologically competent cell types such as primary bone marrow-derived macrophages (BMDMs) (40–42). Using this orthologous platform to evaluate sarbecovirus accessory protein function, we found that 7a promotes viral replication and viral output while inducing proinflammatory responses that are mediated, in part, through 7a cytoplasmic tail interactions. Subsequent to these studies, we validated findings in recombinant SARS-CoV-2 infection contexts, demonstrating that 7a cytoplasmic tail modifications reduce virus growth in mouse infection models.

## RESULTS

### Construction of recombinant embecoviruses expressing sarbecovirus accessory 7a genes

**Fig 1:**
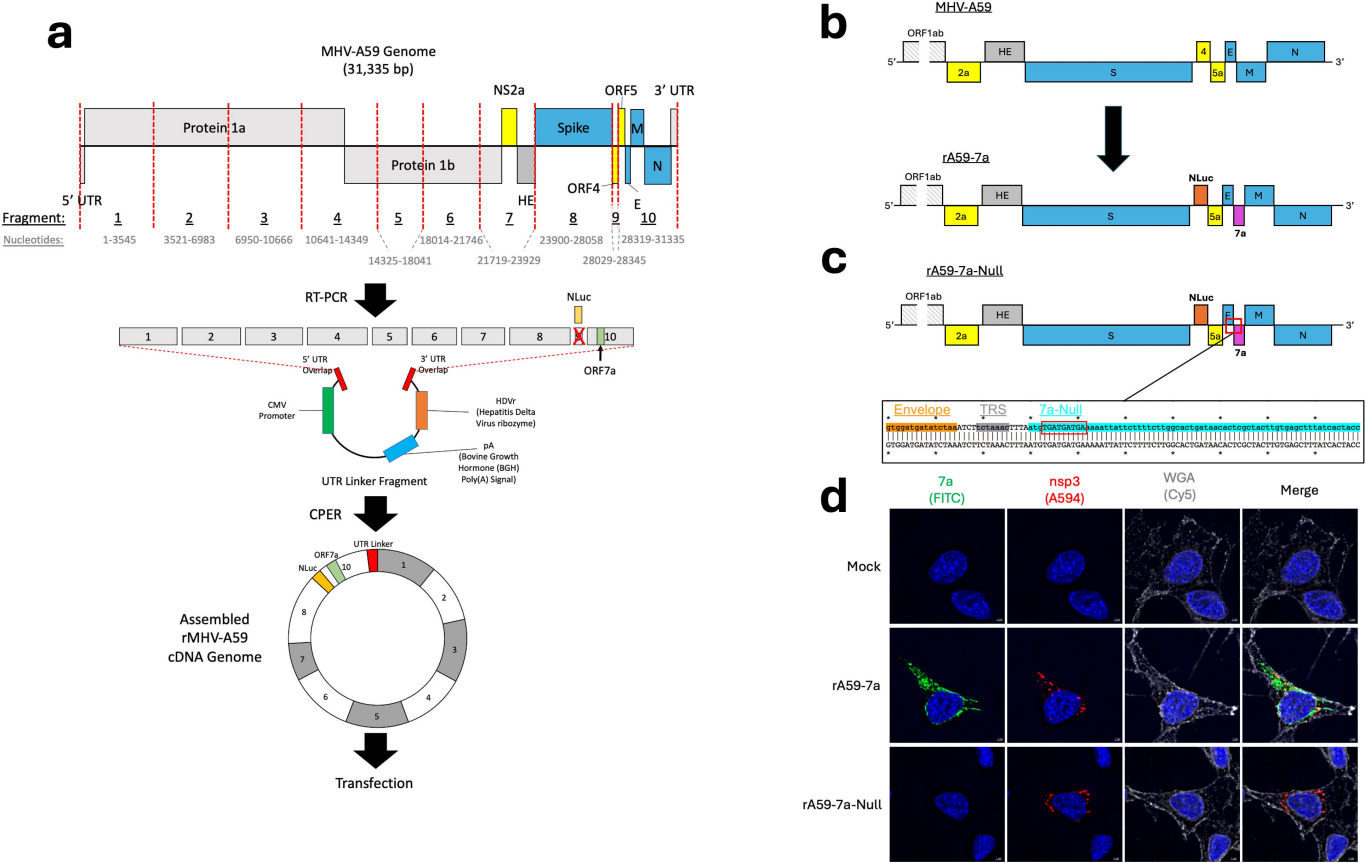
Construction of recombinant embecoviruses expressing sarbecovirus accessory 7a genes. (**a**) A diagram of the mouse hepatitis virus strain A59 (GenBank accession no. AY700211) genome and the circular polymerase extension reaction (CPER) method used for recombinant virus generation. (**b**) The proposed design for rA59-7a. ORF4 was replaced with a nanoluciferase reporter, and ORF7a was inserted, with its own transcription regulatory sequence (TRS), immediately downstream of ORF E. (**c**) The proposed design for the isogenic control, rA59-7a-Null. rA59-7a-Null had an identical design apart from three stop codons inserted immediately downstream of the ORF7a start codon. (**d**) Representative immunofluorescence images of 17Cl-1 mouse fibroblast cells at 8 h post-infection. The cells were infected with the indicated virus at a multiplicity of infection of 1, then probed for SARS-CoV-2 7a in the FITC channel and MHV-A59 nsp3 in the A594 channel. Cell membranes were stained with wheat germ agglutinin (WGA) conjugated with a Cy5 fluorophore. The DAPI stain is shown in blue. The images were taken at ×100 magnification. Scale bars represent 1 µm.

To investigate potential functions of SARS-CoV-2 accessory protein 7a in the context of an orthologous and biosafe betacoronavirus infection, we used reverse genetics and circular polymerase extension reaction (CPER) methodologies (43, 44) to generate a recombinant MHV (strain A59) containing SARS-CoV-2 ORF7a, designated as rA59-7a (Fig. 1a). ORF7a was inserted directly downstream of ORF E, under the control of a dedicated transcription regulatory sequence to ensure subgenomic 7a RNA synthesis (45, 46) (Fig. 1b). In the rA59-7a genome, ORF4 was replaced with a nanoluciferase (Nluc) reporter gene, thereby allowing Nluc enzyme activities to serve as proxies for viral replication. Isogenic control rA59-7a-Null viruses were constructed by introducing three stop codons downstream of the ORF7a start codon (Fig. 1c). The recombinant viruses were generated within HEK293 cells transfected with CPER-amplified DNAs, with secreted viruses then harvested, plaque-purified, and grown up to stock titers on mouse delayed brain tumor (DBT) astrocytoma cells.

Sequence-verified rA59-7a viruses were then used to infect 17Cl-1 mouse fibroblast cells. The infected cells were fixed and stained for immunofluorescence (IF) imaging at 8 h post-infection (hpi). Infected cells were identified by staining for replication organelles using an anti-nsp3 antibody, and 7a expression was assessed with its respective antibody. The results showed abundant 7a expression in the rA59-7a infected cells but none in rA59-7a-Null (Fig. 1d).

### 7a does not antagonize antiviral interferon during rA59 infection

**Fig 2:**
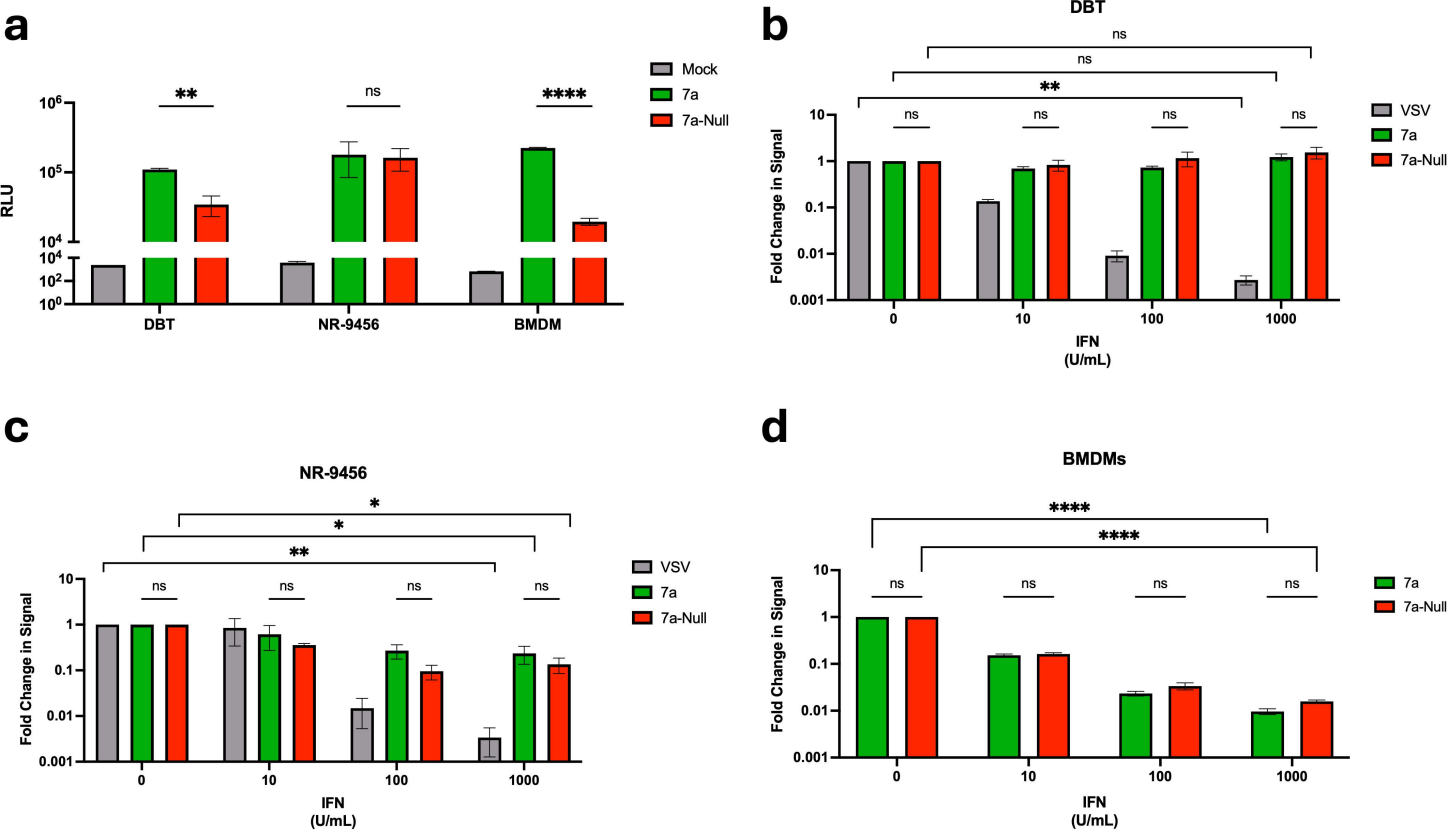
7a does not antagonize antiviral interferon effects during rA59 infection. (**a**) Mouse delayed brain tumor (DBT) cells, immortalized mouse macrophages (NR-9456), and primary mouse bone marrow-derived macrophages (BMDMs) were infected with the indicated virus at a multiplicity of infection (MOI) of 0.1. Infected cells were incubated with Endurazine Live Cell Substrate, and the results were read on a luminometer at 12 hpi for the DBT and NR-9456 cells and 16 hpi for the mouse BMDMs. The results are depicted as the raw relative light unit (RLU) values. Error bars present standard errors of the mean (SEMs) from three biological replicates. Values were analyzed using an unpaired *t*-test. (**b**) Mouse DBT cells were pre-treated with 10-fold titrations of universal type I IFN for 8 h prior to infection with the indicated virus at an MOI of 0.1. Infected cells were incubated with Endurazine Live Cell Substrate, and the results were read on a luminometer at 12 hpi. The results are depicted as fold change in luciferase signal relative to the 0 U/mL IFN control. The error bars present SEM from three biological replicates. Plotted values were analyzed using a two-way ANOVA test. (**c**) NR-9456 mouse macrophages were pre-treated with 10-fold titrations of universal type I IFN for 8 h prior to infection with the indicated virus at an MOI of 0.1. Infected cells were incubated with Endurazine Live Cell Substrate, and the results were read on a luminometer at 12 hpi. The results are depicted as fold change in luciferase signal relative to the 0 U/mL IFN control. The error bars present SEM from three biological replicates. Plotted values were analyzed using a two-way ANOVA test. (**d**) Mouse BMDMs were pre-treated with 10-fold titrations of universal type I IFN for 8 h prior to infection with the indicated virus at MOI 0.1. Infected cells were incubated with Endurazine Live Cell Substrate, and the results were read on a luminometer at 16 hpi. The results are depicted as fold change in luciferase signal relative to the 0 U/mL IFN control. The error bars present SEM from three biological replicates. Plotted values were analyzed using a two-way ANOVA test. ns, *P* > 0.05; *, *P* ≤ 0.05; **, *P* ≤ 0.01; ***, *P* ≤ 0.001; ****, *P* ≤ 0.0001.

SARS-CoV-2 accessory protein 7a was previously shown to function as an interferon antagonist by preventing STAT2 phosphorylation (30–32). These findings were obtained from experiments in which the 7a proteins were expressed from plasmid DNAs in the absence of a complete coronavirus infection. Here we considered whether 7a proteins might increase the known interferon antagonizing potential that is evident in authentic MHV-infected cells (47, 48). To this end, mouse DBT cells were exposed to serially diluted type I interferon 8 h prior to rA59 infection. At 12 hpi, viral Nluc accumulations were compared between rA59-7a and rA59-7a-Null (Fig. 2a). A proviral 7a effect was observed in mouse DBT cells and mouse BMDMs, but no significant difference was observed in an immortalized mouse macrophage cell line (NR-9456). Normalized data processing was used to evaluate effects of interferon (Fig. 2b). Surprisingly, interferon treatments did not suppress rA59-7a or rA59-7a-Null infections. By contrast, VSV infections were clearly dose-dependently suppressed, indicating intact antiviral interferon signaling in the DBT cells. The experiment was repeated in immortalized mouse macrophages and in IFN-sensitive primary mouse BMDMs. In these settings, interferon equally suppressed both rA59-7a and rA59-7a-Null due to interferon treatment, up to 10-fold and 100-fold in the immortalized and primary macrophages, respectively (Fig. 2c and d). These results demonstrate that 7a did not operate to antagonize the anti-MHV activities of type I interferon in murine cells, affording an opportunity to further dissect IFN-independent 7a activities.

### 7a increases virus replication and viral titers

**Fig 3:**
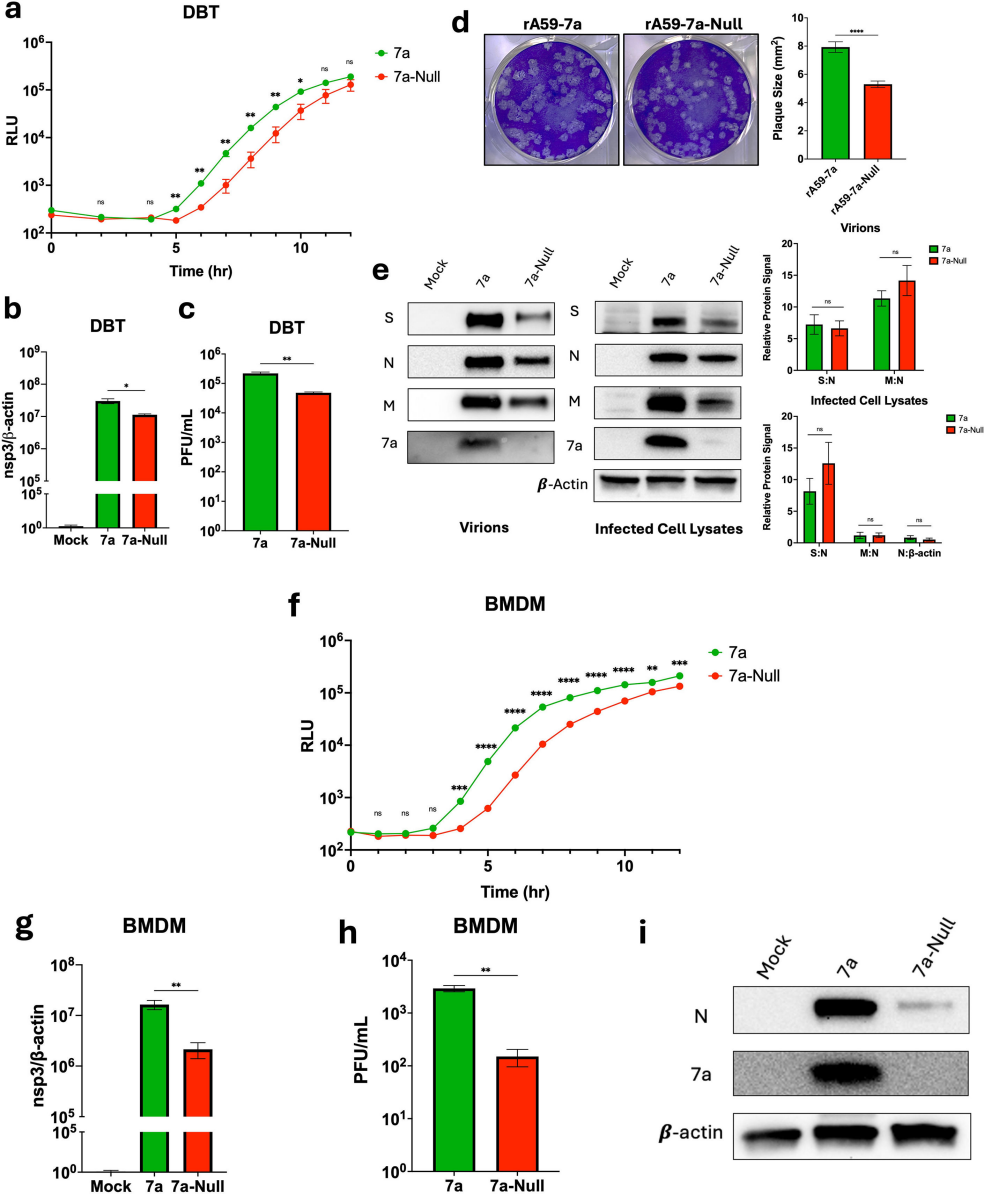
7a increases virus replication and viral titers. (**a**) An infection time course assay, where mouse DBT cells were infected with the indicated virus at an MOI of 0.1. Infected cells were incubated with Endurazine Live Cell Substrate, and the results were read on a luminometer over 12 h. The data are shown as relative light units (RLU). The error bars present standard errors of the mean (SEMs) from three biological replicates. Plotted values were analyzed using multiple *t*-tests. (**b**) An RT-qPCR assay using RNA isolated at 12 hpi from mouse DBT cells infected at an MOI of 0.1. The RNA was analyzed for relative viral genomes by measuring MHV nsp3. The levels of viral RNA relative to β-actin were expressed as 2^−∆∆Ct^. The error bars present SEM from three biological replicates. Plotted values were analyzed using a one-way ANOVA test. (**c**) The plotted values of plaque assay titers obtained from the virus supernatant collected at 12 hpi at an MOI of 0.1 mouse DBT cell infections. The error bars present SEM from three biological replicates. Plotted values were analyzed using an unpaired *t*-test. (**d**) Plaque assay images from the titered viral supernatant that was collected at an MOI of 0.1 infected mouse DBT cells at 16 hpi. Plaque sizes were quantified using ImageJ. Plotted values were analyzed using an unpaired *t*-test. (**e**) Mouse DBT cells were infected with the indicated virus at MOI 0.1. Virus supernatant and cell lysates were collected at 12 hpi. Concentrated virus supernatants, which were pelleted through 20% (wt/wt) sucrose and resuspended, and infected cell lysates were evaluated by Western blotting. The Western blot assays detected S, N, M, 7a, and β-actin. The protein band signals were quantified using AlphaView. The plotted values represent the average protein signal relative to nucleocapsid or β-actin. Plotted values were analyzed using an unpaired *t*-test. (**f**) An infection time course assay, where mouse bone marrow-derived macrophages (BMDMs) were infected with the indicated virus at an MOI of 0.1. Infected cells were incubated with Endurazine Live Cell Substrate, and the results were read on a luminometer over 12 h. The data are shown as RLU. The error bars present SEM from three biological replicates. Plotted values were analyzed using multiple *t*-tests. (**g**) A RT-qPCR assay using RNA isolated at 16 hpi from mouse BMDMs infected at an MOI of 0.1. The RNA was analyzed for relative viral genomes by measuring MHV nsp3. The levels of viral RNA relative to β-actin were expressed as 2^−∆∆Ct^. The error bars present SEM from three biological replicates. Plotted values were analyzed using a one-way ANOVA test. (**h**) The plotted values of plaque assay titers obtained from the virus supernatant collected at 16 hpi at an MOI of 0.1 mouse BMDM infections. The error bars present SEM from three biological replicates. Plotted values were analyzed using an unpaired *t*-test. (**i**) Mouse BMDMs were infected with the indicated virus at an MOI of 0.1. Cell lysates were collected at 16 hpi and then evaluated by Western blotting. The Western blot assays detected N, 7a, and β-actin. ns, *P* > 0.05; *, *P* ≤ 0.05; **, *P* ≤ 0.01; ***, *P* ≤ 0.001; ****, *P* ≤ 0.0001.

We sought further characterization of the protein 7a-dependent increases in viral RNA replication (Fig. 2a). Experiments were initiated using the DBT cells that did not elicit interferon-induced rA59 restriction. Infected DBT cells were incubated with a live cell nanoluciferase substrate and read at hourly intervals by luminometry. rA59-7a replication began ~1 h earlier than rA59-7a-Null, with the two parallel infections then accumulating Nluc similarly (Fig. 3a). The earlier onset of rA59-7a replication was consistent with RT-qPCR measurements showing higher levels of 7a-positive viral genomes at 12 hpi (Fig. 3b). The findings also aligned with measurements of secreted rA59 viruses, revealing that 7a promoted ~10-fold increases in virus infectivity (Fig. 3c). Supporting 7a proviral activity, the rA59-7a plaques were significantly larger than rA59-7a-Null (Fig. 3d). To further assess 7a-dependent effects on infection, pelleted rA59 virus particles were resuspended, and virion proteins were detected by Western blotting. rA59-7a virion yields were clearly higher than rA59-7a-Null, as evidenced by S, N, and M band intensities (Fig. 3e). 7a incorporated into MHV virions, as might be predicted from evaluations of SARS-CoV (16), but did not alter the virion S:N or M:N ratios (Fig. 3e). The increased yields of secreted rA59-7a virion proteins concorded with increased virion proteins in cell lysates (Fig. 3e), suggesting that 7a promotes viral replication or translation but may not specifically augment virion assembly or secretion processes.

Similar experiments were performed using primary BMDMs. rA59 can be restricted in BMDMs (Fig. 2), and primary BMDMs are relevant *ex vivo* models for MHV–host cell interactions (40, 41, 49). Furthermore, SARS-CoV-2 infection of macrophages, while abortive (50–53), elicits disease-relevant cytokine responses (54–56), making BMDMs a reasonable choice for further evaluation of 7a phenotypes. Infected BMDMs were monitored for Nluc reporter accumulations. Again, rA59-7a replication advanced earlier than 7a-Null, here about 2 h earlier (Fig. 3f). Relative to rA59-7a-Null, the rapid rA59-7a replication onset generated ~10-fold more viral genomes at 16 hpi (Fig. 3g), ~20-fold more secreted virion infectivities (Fig. 3h), and significantly more intracellular N proteins (Fig. 3i). These findings matched those obtained using DBT cells.

### Substitutions in the 7a cytoplasmic tail reduce proviral activities

The 121-aa transmembrane 7a proteins have short cytoplasmic tails (K_117_RKTE_121_) that include canonical di-lysine ER retention motifs (KXKXX). Host-cell protein trafficking machinery, such as the COPI coatomer complex, interacts with these motifs and likely retains 7a to the ER and ER–Golgi intermediate compartment (20, 29, 57, 58), the membrane organelles essential for viral replication (59, 60) and viral assembly (61–63). Specifically, the K117 and K119 residues are considered essential for COPI coatomer binding (64).

Additionally, the K119 residue has been implicated in ubiquitination (26, 27, 30) and subsequent host factor binding (27), potentially augmenting interactions with host factors and other viral structural components (16). Therefore, to assess the relevance of the cytoplasmic motif, recombinant viruses were constructed in which each lysine (K117 or K119) was substituted with an alanine. The K117A and K119A recombinant viruses were designated rA59-7a-ARKTE and rA59-7a-KRATE, respectively.

**Fig 4:**
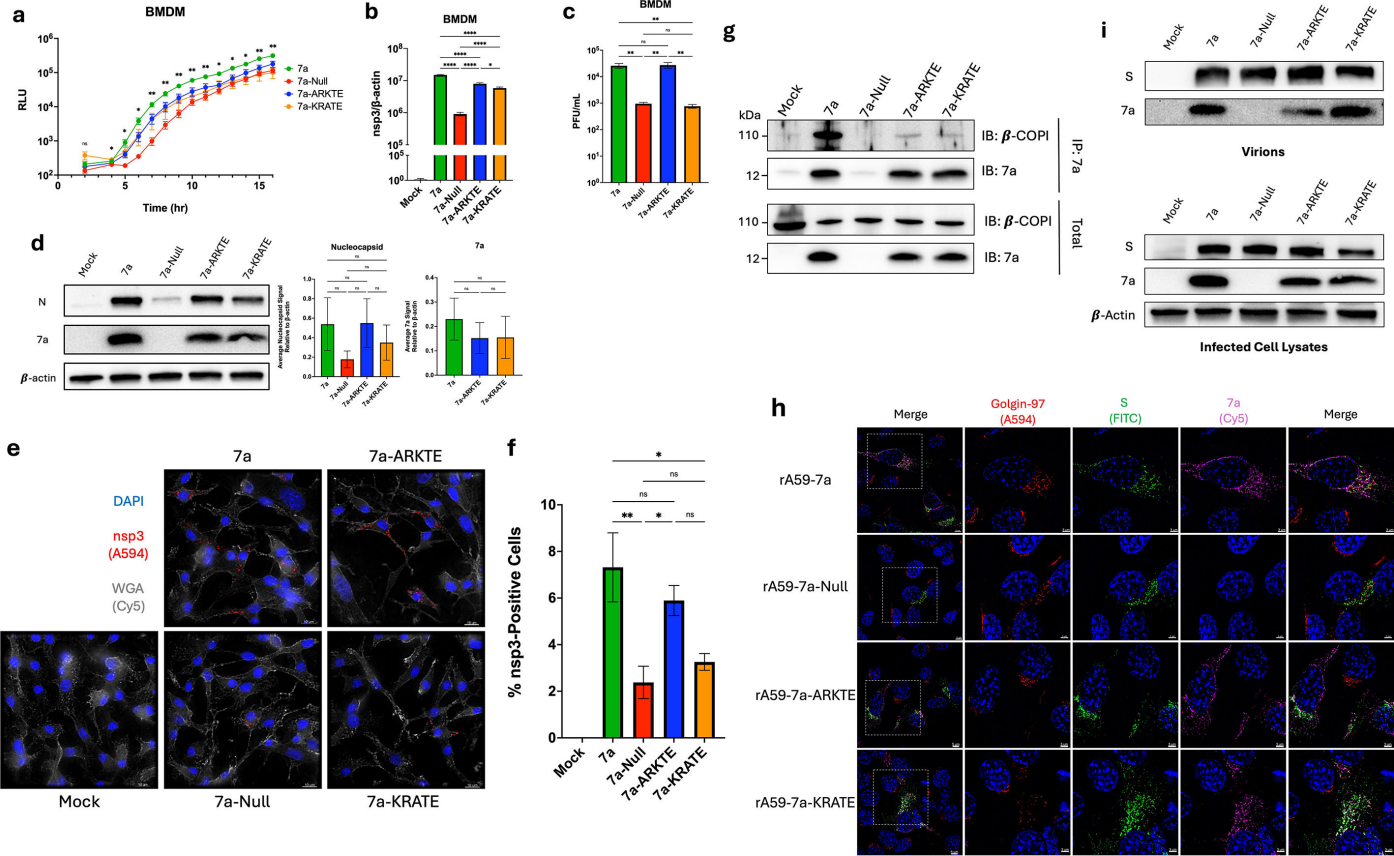
Substitutions in the 7a cytoplasmic tail reduce proviral activities. (**a**) An infection time course assay, where BMDMs were infected with the indicated viruses at an MOI of 0.1. Infected cells were incubated with Endurazine Live Cell Substrate, and the results were read on a luminometer over 16 h. The data are shown as relative light units (RLU). Error bars present standard errors of the mean (SEMs) from three biological replicates. Plotted values were analyzed using a one-way ANOVA test. (**b**) An RT-qPCR assay using RNA isolated at 16 hpi from mouse BMDMs infected at an MOI of 0.1. The RNA was analyzed for relative viral genomes by measuring MHV nsp3. The levels of viral RNA relative to β-actin were expressed as 2^−∆∆Ct^. The error bars present SEM from three biological replicates. Plotted values were analyzed using a one-way ANOVA test. (**c**) The plotted values of plaque assay titers from virus supernatant collected at 16 hpi at an MOI of 0.1 mouse BMDM infections. The error bars present SEM from eight biological replicates. Plotted values were analyzed using an unpaired *t*-test. The data shown are an aggregate of three independent experiments. (**d**) Mouse BMDMs were infected with the indicated virus at an MOI of 0.1. Cell lysates were collected at 16 hpi and then evaluated by Western blot. The Western blot assays detected N, 7a, and β-actin. The protein band signals were quantified using AlphaView. The plotted values represent the average protein signal relative to β-actin. Plotted values were analyzed using a one-way ANOVA test. (**e**) Representative IF images of mouse BMDMs at 6 hpi. Cells were infected with the indicated virus at an MOI of 0.1. MHV-A59 nsp3 was probed in the A594 channel. Cell membranes were stained with WGA conjugated with a Cy5 fluorophore. DAPI stain is shown in blue. Images were taken at ×60 magnification. Scale bars represent 10 µm. (**f**) The quantification of nsp3-positive BMDMs from IF images. The error bars present SEM from five biological replicates. Plotted values were analyzed using a one-way ANOVA test. (**g**) Mouse DBT cells were infected with the indicated virus at an MOI of 0.1, then lysed with 1% NP-40 lysis buffer at 16 hpi, followed by IP using anti-7a monoclonal antibody-bound Dynabeads. The immunocomplex was assessed via Western blot to detect β-COPI and 7a. (**h**) Representative high-resolution IF images of 17Cl-1 mouse fibroblast cells at 10 hpi that were obtained using structural illumination microscopy. Cells were infected with the indicated virus at an MOI of 1. Golgin-97, a trans-Golgi (TGN) marker, was probed in the A594 channel; MHV-A59 S was probed in the FITC channel; and SARS-CoV-2 7a was probed in the Cy5 channel. DAPI stain is shown in blue. Images were taken at ×100 magnification. Scale bars represent 3 µm. (**i**) Mouse DBT cells were infected with the indicated virus at an MOI of 0.1. Virus supernatant and cell lysates were collected at 16 hpi. Concentrated virus supernatants, which were pelleted through 20% (wt/wt) sucrose and resuspended, and infected cell lysates were evaluated by Western blotting. The Western blot assays detected S, 7a, and β-actin. ns, *P* > 0.05; *, *P* ≤ 0.05; **, *P* ≤ 0.01; ***, *P* ≤ 0.001; ****, *P* ≤ 0.0001.

Mouse BMDMs were inoculated with the mutant viruses, and replication kinetics were monitored through a single infection cycle. rA59-7a-ARKTE and rA59-7a-KRATE advanced at rates that were intermediate between the fast rA59-7a and the slow A59-7a-Null (Fig. 4a). Viral genome levels at 16 hpi were concurrently modestly decreased relative to rA59-7a (Fig. 4b). Secreted virus rA59-KRATE infectivities were expectedly low; however, there was a puzzling high rA59-ARKTE infectivity yield that was discordant with the reduced replication of this variant (Fig. 4c). rA59-7a-KRATE-infected cells contained viral nucleocapsid proteins at levels intermediate between the respective high- and low-output 7a and 7a-Null infected cells (Fig. 4d). To further assess the effect of 7a cytoplasmic tail substitutions on viral replication, infected BMDMs were fixed and probed for viral replication organelles using an anti-nsp3 antibody (Fig. 4e). Relative to the rA59-7a infections, there were significantly decreased numbers of BMDMs with established replication organelles in rA59-7a-KRATE-infected cultures (Fig. 4f). For the most part, these results reflect the measurements of viral genome accumulations through 16 h (Fig. 4b), although there was some discordance with the high rA59-7a-KRATE genome levels. The results suggest that lysine substitutions in 7a cytoplasmic tails reduce 7a proviral activities and that proviral effects take place at early RNA replication stages. The findings also leave open the possibility that the K119 residue, a known ubiquitin substrate (26, 27, 30), also facilitates virus assembly or egress (Fig. 4c).

While it has been assumed that the COPI coatomer complex interacts with the cytoplasmic ER retention motif of 7a, a direct interaction has not been shown. Therefore, 7a proteins were immunoprecipitated (IP) from infected DBT cell lysates. IP of 7a from the rA59-7a infection co-precipitated the β-COPI subunit of the coatomer complex (65, 66), while IP of 7a-ARKTE and 7a-KRATE precipitated significantly less β-COPI (Fig. 4g). High-resolution IF images were taken using structured illumination microscopy (SIM) to assess potential effects of the reduced coatomer interactions on 7a localization. 7a, 7a-ARKTE, and 7a-KRATE all partially co-localized with viral S proteins and also with Golgin-97, a Golgi resident protein (Fig. 4h). Thus, surprisingly, disruption of the 7a–COPI interaction did not significantly alter 7a intracellular localization during MHV infection. This raised questions of whether 7a incorporation into virions was affected by the reduced COPI binding. Western blot analysis of secreted virion proteins revealed that the KRATE variant was present in the virion protein preparations in abundance similar to wild type 7a, while the ARKTE variant 7a showed less virion incorporation (Fig. 4i). The results demonstrated that these substitutions in the 7a cytoplasmic tail reduced interactions with coatomer complexes but had variable and somewhat marginal effects on 7a intracellular localization and virion incorporation.

### 7a mediates a proinflammatory response in BMDMs that depends on the cytoplasmic tails

**Fig 5:**
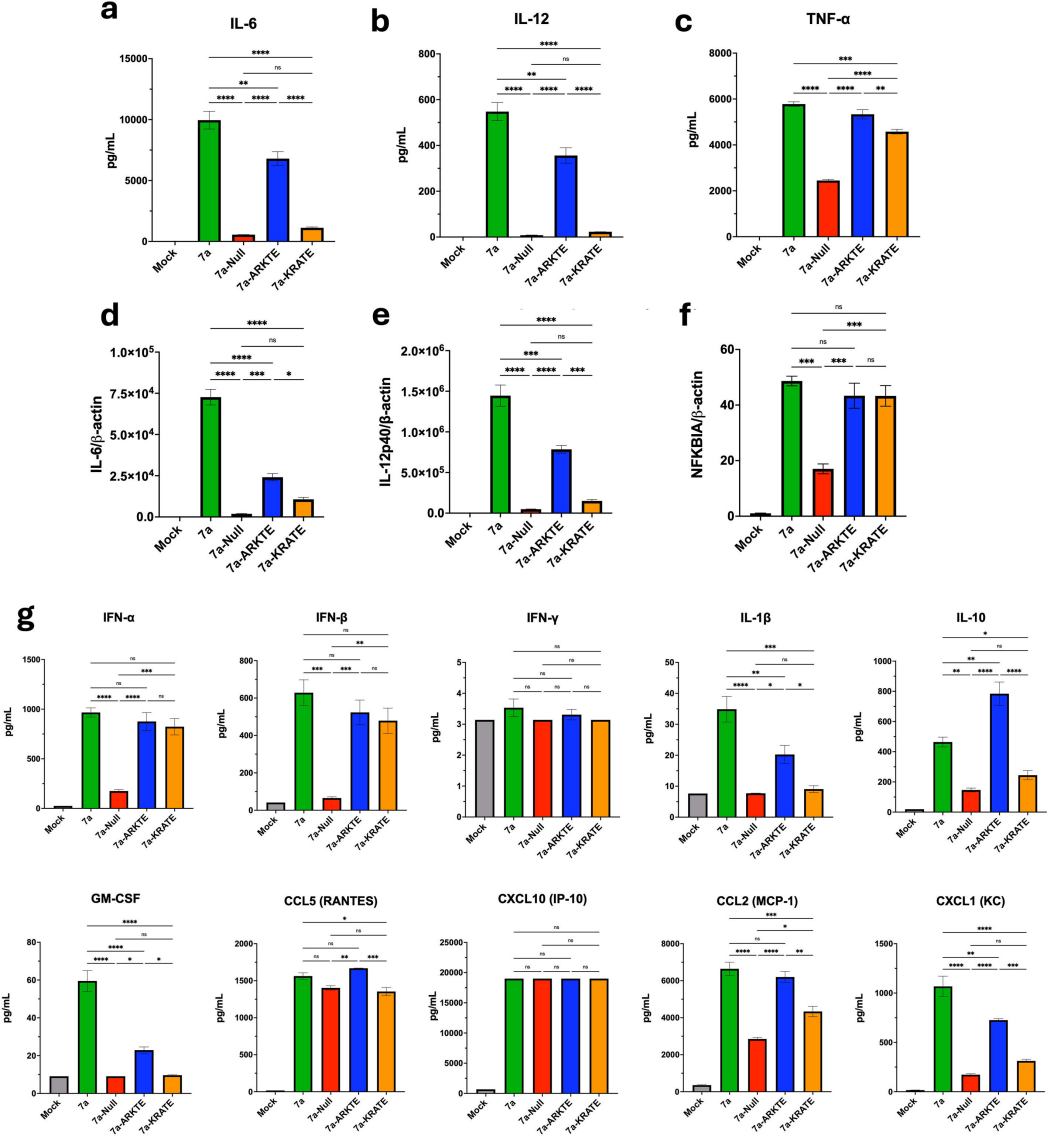
7a mediates a proinflammatory response in BMDMs that depends on the cytoplasmic tails. (**a–c**) Clarified virus supernatants at an MOI of 0.1 infected mouse BMDMs were collected at 16 hpi and assessed by a LEGENDplex Mouse Anti-Virus response panel to quantify IL-6, IL-12, and TNF-α proinflammatory cytokine levels. The error bars present standard errors of the mean (SEMs) from three biological replicates. Plotted values were analyzed using a one-way ANOVA test. (**d–f**) An RT-qPCR assay using RNA isolated at 16 hpi from mouse BMDMs infected at an MOI of 0.1. The RNA was analyzed for relative IL-6, IL-12p40, and NFKBIA gene expression levels. The gene expression levels relative to β-actin were expressed as 2^−∆∆Ct^. The error bars present SEM from three biological replicates. Plotted values were analyzed using a one-way ANOVA test. (**g**) Additional plots of cytokine levels assessed by the LEGENDplex Mouse Anti-Virus response panel. The error bars present SEM from three biological replicates. Plotted values were analyzed using a one-way ANOVA test. ns, *P* > 0.05; *, *P* ≤ 0.05; **, *P* ≤ 0.01; ***, *P* ≤ 0.001; ****, *P* ≤ 0.0001.

Several previous studies have documented that accessory protein 7a can promote proinflammatory cytokine production (27, 28, 32), potentially mediated by the 7a cytoplasmic tails, specifically K119 (27), and operating through the NF-κB pathway (27, 28). This suggests that 7a contributes to the elevated levels of proinflammatory cytokines, such as IL-6 and IL-12, observed in sera obtained from severe COVID-19 patients (67). We considered whether the various rA59-7a infections might generate differential proinflammatory responses and, if so, whether there are correlations between proinflammatory cytokines and virus replication and secreted virus yield. To this end, conditioned media from infected BMDMs were evaluated using a LEGENDPlex Mouse Anti-Virus Response panel kit (Fig. 5). rA59-7a and rA59-7a-ARKTE had significantly greater levels of IL-6 and IL-12 compared to rA59-7a-Null and rA59-7a-KRATE (Fig. 5a and b). These findings aligned with cytokine RNA levels, as measured by RT-qPCR (Fig. 5d and e), indicating controls at signaling and cytokine gene expression, not at the latest stages of cytokine protein secretion from BMDMs. The decreased IL-6 and IL-12 levels for rA59-7a-Null and rA59-7a-KRATE strongly correlated with their decreased viral titers, suggesting a link between proinflammatory cytokine production and virus secretion.

TNF-α cytokine and *NFKBIA* gene expression levels were assessed to determine if the 7a-dependent proinflammatory response observed in infected BMDMs was due to activation of the NF-κB pathway. TNF-α cytokine production and *NFKBIA* gene expression are common correlates for NF-κB activation (27, 28, 68). TNF-α levels were significantly decreased in rA59-7a-Null infections but only slightly decreased in the parallel rA59-7a-ARKTE and rA59-7a-KRATE-infected cells (Fig. 5c). Relative to rA59-7a, *NFKBIA* gene expression was significantly decreased only in the rA59-7a-Null infected cells (Fig. 5f). The results suggest that the NF-κB pathway may be activated by 7a, without the essential participation of the K117 or K119 residues of the cytoplasmic tail. Yet the K residues, most notably K119, appear to have a significant role in promoting IL-6 and IL-12 proinflammatory cytokine production by infected BMDMs in an NF-κB independent manner.

### The 7a K119A substitution attenuates mouse-adapted SARS-CoV-2 infections

**Fig 6:**
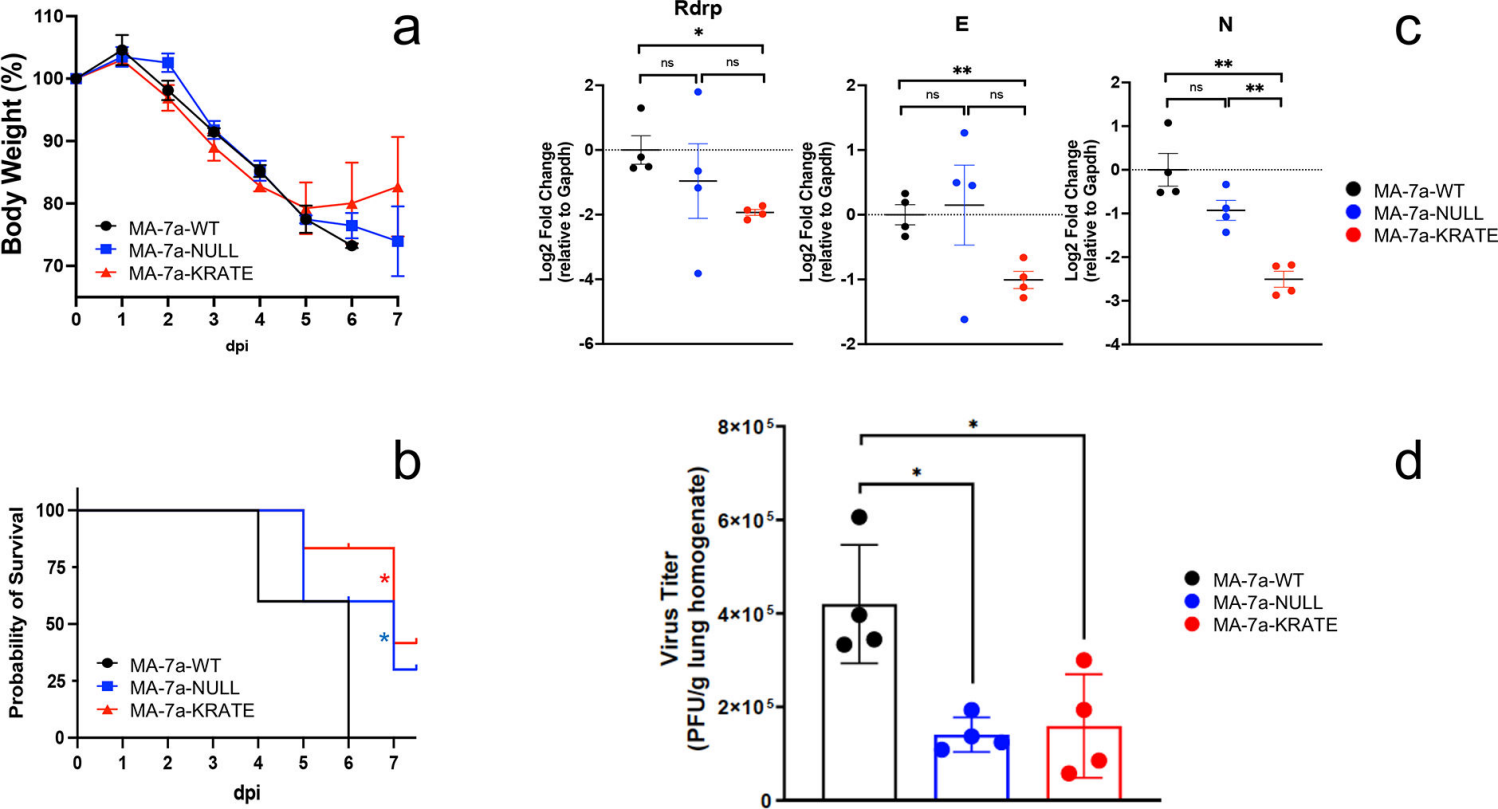
Substitutions in the 7a cytoplasmic tail reduce mouse-adapted SARS-CoV-2 replication and pathogenesis in mice. (**a–c**) Ten-week-old BALB/c mice were intranasally inoculated with 1,000 PFU or (**d**) 500 PFU of the indicated MA viruses (*n* = 4–5 mice for each virus infection). (**a**) Mice were monitored for weight loss and (**b**) humane endpoints. Plotted values in (**b**) were analyzed using a log-rank test (*P* = 0.0414). (**c**) Viral RNA accumulations in lungs were quantified at 3 dpi by RT-qPCR. (**d**) Virus titers were determined by plaque assay on VeroE6-TMPRSS2 indicator cells. (**c and d**) Each data point represents a biological replicate measured in duplicate. Plotted values were analyzed using an unpaired Student’s *t*-test (ns, *P* > 0.05; *, *P* ≤ 0.05; **, *P* ≤ 0.01 ).

To determine whether findings in the rA59 virus background are similarly observed in SARS-CoV-2 infections, we introduced stop codons and K119A codon changes into a mouse-adapted (MA) SARS-CoV-2 bacterial artificial chromosomal plasmid (BACmid) using fragment assembly methods. Transfection of the resulting recombinant BACmids into Vero-ACE2-TMPRSS2 cells generated a set of three recombinant SARS-CoV-2 viruses: unaltered MA (MA-7a-WT), MA-7a-NULL, and MA-7a-KRATE. These viruses were used to infect susceptible BALB/c mice. In each of the three infections, weight losses were similar, although some recovery from MA-7a-KRATE infections was evident at 6–7 dpi (Fig. 6a). Both the elimination of the 7a expression (MA-7a-NULL) and the 7a-K119A substitution (MA-7a-KRATE) significantly attenuated the infections as measured by survival scores (Fig. 6b). This attenuation accorded with viral RNA levels in lungs at 3 dpi; mice infected with MA-7a-KRATE had two- to sixfold less RNA accumulation than MA-7a-WT (Fig. 6c). Correspondingly, MA-7a-NULL and MA-7a-KRATE viral titers were significantly lower at 3 dpi (Fig. 6d). These findings further demonstrate that residue K119 in the 7a cytoplasmic tail supports coronavirus infection and validates the results observed in an rA59 virus background.

## DISCUSSION

Severe COVID-19 is often correlated with a dysregulated release of proinflammatory cytokines. Patients have elevated levels of IL-6, IL-12, IL-1β, and other proinflammatory cytokines that contribute to infection clearance but at high levels cause severe, even fatal disease (67). SARS-CoV-2-infected macrophages have been implicated as key drivers of inflammatory cytokines and resultant severe COVID-19 (54–56). However, consistent *in vitro* model systems for SARS-CoV-2 macrophage infection have yet to be established (50–53), as was the case for SARS-CoV (69, 70). MHV productively infects BMDMs *ex vivo* due to their high expression of the MHV receptor murine carcinoembryonic antigen-related cell adhesion molecule 1 (CEACAM1) (42, 56). This BMDM susceptibility provides a premise to use recombinant MHVs as vectors to express SARS-CoV-2 genes in betacoronavirus infection contexts. We constructed recombinant MHVs expressing the SARS-CoV-2 accessory gene 7a and infected murine BMDMs. We discovered that SARS-CoV-2 protein 7a was a likely contributor to the elevated proinflammatory response that occurs during infection. Recombinant MHV A59-7a infections elicited IL-6 and IL-12 production in BMDMs. Cytokine release was linked to virus secretion through an unknown mechanism requiring native 7a cytoplasmic tails, specifically 7a tail residue K119. It remains unclear how 7a cytoplasmic tails manipulate the host cell to promote virus secretion and a proinflammatory response, but the mechanism appears to be independent of the COPI coatomer complex and the NF-κB pathway.

Utilizing recombinant MHVs to infect BMDMs also revealed a role for SARS-CoV-2 accessory protein 7a in promoting viral replication and output. In both murine BMDMs and DBT cells, 7a proteins accelerated the kinetics of viral replication (Fig. 3a and f) and significantly increased secreted virus levels (Fig. 3c and h). These 7a-dependent proviral effects were independent of a reported 7a-mediated interferon antagonizing activity (30–32). Indeed, in our tests, 7a did not antagonize antiviral interferon responses (Fig. 2). The proviral effects of 7a were diminished by substituting cytoplasmic tail lysine residues, particularly K119, whose exchange to A reduced viral titers. The mechanisms by which the cytoplasmic tails elicit proviral effects remain unknown. Substitutions in SARS-CoV-2 7a cytoplasmic tails have been shown to relocalize host factors, particularly to affect factors influencing MHC-I localization (20), but we did not find that mutant 7a proteins were significantly relocalized in infected cells (Fig. 4h). Relocalized 7a may not have been detectable due to MHV infection-induced disruption of the secretory pathway; note, for example, the faint, scattered Golgin-97 signal in SIM images (Fig. 4h). 7a proteins may interfere with cellular autophagy (23, 24). As coronaviruses are thought to egress through an autophagosome–lysosome pathway (71), 7a may facilitate virus flow through secretory autophagosomes. Similar proviral effects have been reported for SARS-CoV-2 accessory protein 3a, which manipulated autophagy and modulated intracellular trafficking through its cytoplasmic tail to promote viral output (14, 24, 72, 73). Future studies are aimed at determining whether 7a promotes replication by modifying the autophagosomal organelles supporting coronavirus replication and assembled virus secretion (74, 75).

This study establishes the utility of a recombinant MHV system to analyze individual SARS-CoV-2 proteins. Genes and proteins of pathogenic human coronaviruses can be evaluated at BSL-2 when placed in the MHV background. This was needed for the 7a study, as the proviral role for 7a required analyses in the complete viral infection context. The MHV system addresses functional redundancies among sarbecovirus accessory proteins; notably, the deletion of ORF7a in SARS-CoV-2 generated very minor effects on viral replication (4, 8, 9), possibly due to compensating SARS-CoV-2 gene products (14, 24, 72, 73). MHV only encodes three accessory proteins, ORF2a, ORF4, and ORF5a, which lack homology with SARS-CoV-2 accessory proteins (35–37). The distinctly orthologous set of accessory proteins in MHV permits it to be used as a controlled recombinant virus platform for isolating the effects of particular SARS-CoV-2 accessory proteins.

The non-essential SARS-CoV-2 accessory proteins impact virulence by controlling innate immune responses and by promoting particular stages of virus production (1, 2, 4–6, 14, 15, 31). This study highlighted these attributes of accessory proteins by revealing a proviral effect of SARS-CoV-2 7a during infection of primary innate immune cells and mice. Specifically, 7a promoted viral output and proinflammatory responses through a process requiring native 7a cytoplasmic tails. The importance of the native 7a cytoplasmic tail was further recognized in SARS-CoV-2 infection contexts. These effects on virus production require additional investigation because they appear to be unrelated to activities previously assigned to the 7a cytoplasmic tails, including coatomer-mediated protein trafficking and suppression of interferon signaling (20, 30). Additional studies will also be necessary to dissect the proviral mechanisms by which SARS-CoV-2 accessory protein 7a contributes to the cytokine storms observed in severe cases of COVID-19.

## MATERIALS AND METHODS

### Cells

HEK293T cells (obtained from Edward M. Campbell, Loyola University Chicago) were cultured in Dulbecco’s modified Eagle medium (DMEM) (#10-013-CV, Corning) supplemented with 0.1 mM non-essential amino acids (Gibco), 2 mM L-glutamine (Cytiva), 10 mM HEPES (Corning), 100 U/mL penicillin (Cytiva), 100 µg/mL streptomycin (Cytiva), and 10% fetal bovine serum (FBS) (Bio-techne). Mouse DBT cells (obtained from Susan Baker, Loyola University Chicago) were cultured in minimum essential medium (MEM) Alpha Medium (#11900-024, Gibco) supplemented with 2.2 g/L NaHCO_3_ (Fisher Chemical), 10% Tryptose Phosphate Broth (BD Biosciences), 2 mM L-Glutamine (Cytiva), 100 U/mL Penicillin (Cytiva), 100 µg/mL Streptomycin (Cytiva), and 5% FBS (Bio-techne). 17Cl-1 mouse fibroblast cells (obtained from Susan Baker, Loyola University Chicago) were cultured in DMEM (#10-013-CV, Corning) supplemented with 2 mM L-Glutamine (Cytiva), 5% Tryptose Phosphate Broth (BD Biosciences), 100 U/mL Penicillin (Cytiva), 100 µg/mL Streptomycin (Cytiva), and 5% FBS (Bio-techne). The mouse macrophage cell line, NR-9456, (obtained from BEI Resources) was cultured in DMEM (#10-013-CV, Corning) supplemented with 0.1 mM non-essential amino acids (Gibco), 2 mM L-Glutamine (Cytiva), 10 mM HEPES (Corning), 100 U/mL Penicillin (Cytiva), 100 µg/mL Streptomycin (Cytiva), and 10% FBS (Bio-techne). L929 mouse fibroblast cells (obtained from Andrew T. Ulijasz, Loyola University Chicago) were cultured in minimum essential medium Eagle (MEM) (#10-010-CV, Corning) supplemented with 2 mM L-glutamine (Cytiva), 1 mM sodium pyruvate (Corning), 10 mM HEPES (Corning), and 10% FBS (Bio-techne). All cell lines were cultured in a 5% CO_2_ incubator at 37°C.

### BMDMs

Male and female black/B6 mouse bone marrow (obtained from Dorothy Sojka, Loyola University Chicago) was pooled then cultured in BMDM media, containing DMEM (#10-017-CV, Corning), 30% L929 cell supernatant, 2 mM L-glutamine (Cytiva), 1 mM sodium pyruvate (Corning), 10 mM HEPES (Corning), 100 U/mL penicillin (Cytiva), 100 µg/mL streptomycin (Cytiva), 3.5 × 10^−4^% 2-mercaptoethanol (Sigma), and 25% FBS (Bio-techne), to obtain differentiated BMDMs. Differentiated BMDMs were maintained in BMDM media (as described above).

### Recombinant virus

Recombinant MHV-A59 viruses were generated using the CPER method as described in Amarilla et al. and Torii et al. (43, 44). Recombinant viral genomes were transfected (LipoD293 Transfectant Reagent, SignaGen) into HEK293T cells and co-cultured with mouse DBT cells. Upon observation of cytopathic effects (CPE), cell supernatant was collected and clarified (centrifugation at 300 × *g* for 10 min, followed by 3,000 × *g* for 10 min at 4°C) as the viral stock. Recombinant viruses were propagated and plaque-purified on mouse DBT cells. The modified genomic sequences of all virus stocks were converted to cDNA via RT-PCR (SuperScript IV One-Step RT-PCR System, Invitrogen) and sequence verified by ACGT. Recombinant MHV sequences were aligned to the murine hepatitis virus strain A59 complete genome (GenBank accession no. AY700211). To obtain purified viral particles, the clarified viral supernatant was concentrated 100-fold through centrifugation by overlaying the viral supernatant onto a 20% (wt/wt) sucrose cushion and performing slow-speed pelleting (SW28; 7,500 rpm at 4°C for 24 h). The resulting pellet was resuspended in serum-free DMEM to 1/100 of the original medium volumes.

### Virus infections

Mouse DBT cells were infected with indicated viral strains at a multiplicity of infection (MOI) of 0.1, unless otherwise indicated, in serum-free DMEM media (#10-013-CV, Corning) for 1 h at 37°C in a 5% CO_2_ incubator. After 1 h, the viral inoculum was rinsed off with two PBS rinses followed by the addition of serum-containing MEMs (as described above). Mouse BMDMs were infected with indicated viral strains at an MOI of 0.1, unless otherwise indicated, in serum-free DMEM media (#10-017-CV, Corning) for 1 h at 37°C in a 5% CO_2_ incubator. After 1 h, the viral inoculum was rinsed off with two PBS rinses followed by the addition of serum-free DMEMs (#10-017-CV, Corning). Cell supernatant was collected and clarified (centrifugation at 300 × *g* for 10 min, followed by 3,000 × *g* for 10 min at 4°C) and used for plaque assay and cytometric bead array (CBA) analysis. Infected cells were utilized for Trizol (Ambion) RNA extraction or to generate cell lysates using 1% NP-40 lysis buffer (#AAJ60766AK, Thermo Scientific), containing 50 mM Tris-HCl (pH 7.4), 150 mM NaCl, 5 mM EDTA, and 1:1,000 Protease Inhibitor Cocktail (#P1860, Sigma-Aldrich).

### Plaque assays

Virus infectivities were determined by plaque assay using mouse DBT cells as indicator cells. Cells were infected with 10-fold serial dilutions of viral samples for 1 h at 37°C, followed by overlaying with a 0.4% (wt/vol) Noble agar (BD Biosciences) and 1% FBS DMEM (#10-013-CV, Corning) mixture. Plates were incubated at 37°C for 48 h and fixed using 3.7% formaldehyde-PBS solution for 30 min. Viral plaques were visualized by staining with 0.1% crystal violet for 15 min.

### Antibodies

MHV S proteins were detected with the R3300 rabbit polyclonal antibody (PAb) that targets the S2 subunit (76). MHV S proteins were also detected using a mCEACAM-Fc construct that was previously generated (77). MHV N proteins were detected with murine monoclonal antibody (MAb) J3.1. MHV M proteins were detected with murine MAb J1.3. The J3.1 and J1.3 MAbs were a generous gift from John Fleming. SARS-CoV-2 7a proteins were detected with murine MAb clone 3C9 obtained from Invitrogen (#MA5-35944). MHV nsp3 proteins were detected using the anti-D3 rabbit PAb, which was a generous gift from the Susan Baker lab (78). The β-COPI subunits were detected with a rabbit PAb obtained from Abcam (#ab2899). Golgin-97 proteins were detected using a rabbit PAb obtained from Invitrogen (#PA5-30048). β-Actin subunits were detected using murine MAb clone AC-15 conjugated with peroxidase obtained from Sigma-Aldrich (#A3854).

### Western blots

Samples in SDS solubilizer (0.0625 M Tris-HCl [pH 6.8], 10% glycerol, 0.01% bromophenol blue, 2% [wt/vol] SDS, and 2% 2-mercaptoethanol) were heated at 95°C for 5 min, electrophoresed through 4%–20% or 8%–16% Mini-PROTEAN TGX Precast Protein Gels (Bio-Rad), transferred to nitrocellulose membranes (Bio-Rad), blocked with TBST-M (25 mM Tris-HCl [pH 7.5], 140 mM NaCl, 27 mM KCl, 0.05% Tween 20, and 5% nonfat milk powder), and incubated with the indicated primary antibody. After incubation with the appropriate horseradish peroxidase-tagged secondary antibody and chemiluminescent substrate (Thermo Scientific), the blots were imaged and processed with a FluorChem E apparatus (ProteinSimple).

### RT-qPCR

RNA was isolated from mouse DBT cells or BMDMs using Trizol (Ambion) via phase separation. RT-qPCR was performed using Luna Universal One-Step RT-qPCR Kit (New England BioLabs) on a CFX Opus 96 Real-Time PCR System (Bio-Rad). qPCR primers are listed in Table 1. Cycle thresholds were normalized to that of the housekeeping gene β-actin by the following the equation ∆∆C_T_ = ∆C_T (gene of interest)_ – ∆C_T (β-actin − average)_. ∆C_T_ values were determined by the following equation: ∆C_T_ = C_T (gene of interest)_ – C_T (β-actin)_. All results are shown as a ratio to β-actin calculated as 2^−∆∆CT^.

**TABLE 1:** Primers used for RT-qPCR

Gene	Forward (5′→3′)	Reverse (5′→3′)
Mouse β-actin	GTGACGTTGACATCCGTAAAGA	GCCGGACTCATCGTACTCC
MHV nsp3	TGAAGGCATTGTGCGTGTTG	TCCTCGGCCTCCTCACATAA
Mouse IL-6	CTTCACAAGTCGGAGGCTTAAT	ACTCCAGGTAGCTATGGTACTC
Mouse IL-12p40	CAGAAGCTAACCATCTCCTGGTTTG	TCCGGAGTAATTTGGTGCTTCACAC
Mouse NFKBIA	GCCAGGAATTGCTGAGGCACTT	GTCTGCGTCAAGACTGCTACAC

### Infection time course luminescence assay

Mouse DBT cells or BMDMs in their respective media (as described above), containing Nano-Glo Endurazine Live Cell Substrate (#N2570, Promega) as per manufacturer’s instructions, were infected with the indicated viral strains at an MOI of 0.1 and incubated at 37°C in a 5% CO_2_ incubator. Relative light unit readings were taken on a GloMax Explorer luminometer (Promega) at the indicated timepoints.

### Interferon treatment assay

Mouse DBT cells, NR-9456 mouse macrophages, or mouse BMDMs were pre-treated with 10-fold titrations of Universal Type I IFN (#11200, PBL Assay Science) in their respective media (as described above) for 8 h prior to infection. After the 8 h IFN treatment, cells were rinsed with PBS and infected as described above, unless otherwise indicated. Infected cells were incubated with Nano-Glo Endurazine Live Cell Substrate (#N2570, Promega), and the results were read on a GloMax Explorer luminometer (Promega) at the indicated timepoints. Results were depicted as fold change in signal relative to the 0 U/mL IFN condition.

### Immunofluorescence assay

Mouse fibroblast 17Cl-1 cells or mouse BMDMs were grown on 12 mm coverslips (#12-545-80, Fisher Scientific) and infected before being fixed with 3.7% paraformaldehyde in PBS at the indicated hpi times. After permeabilization using 0.2% Triton X-100 in PBS and subsequent blocking with 1% goat serum (Gibco) in PBS, proteins of interest were detected using the indicated primary antibodies followed by incubation with secondary antibody conjugated to Alexa 488, Alexa 594, or Alexa 647 fluorophores (Life Technologies). mCEACAM-Fc was directly conjugated with a CF488 fluorophore using the Mix-n-Stain CF488A Antibody Labeling Kit (#MX488AS20, Sigma-Aldrich). Cell membranes were stained with wheat germ agglutinin, Alexa Fluor 647 conjugate (#W32466, Invitrogen). Nuclei were stained using Hoescht 33258 (Molecular Probes). Coverslips were mounted with Fluoro-Gel with Tris Buffer (#17985-10, Electron Microscopy Sciences) onto glass slides for imaging. Fluorescence signals were captured with a DeltaVision wide field fluorescence microscope (Applied Precision, GE) equipped with a digital camera (CoolSNAP HQ, Photometrics) and a 1.4-numerical aperture ×100 objective lens. Z-stack images were collected and deconvolved with SoftWoRx deconvolution software (Applied Precision, GE). High-resolution SIM images were captured with a Lattice SIM5 (Zeiss). Microscopy images were processed using Imaris (Bitplane).

### Immunoprecipitation assay

Mouse DBT cells were infected with indicated viral strains at an MOI of 0.1 in serum-free DMEMs (#10-013-CV, Corning) for 1 h at 37°C in a 5% CO_2_ incubator. After 1 h, the viral inoculum was rinsed off with two PBS rinses followed by the addition of serum-containing MEMs (as described above). At 16 hpi, infected cells were lysed using 1% NP-40 lysis buffer (#AAJ60777AK, Thermo Scientific) containing 50 mM Tris-HCl (pH 7.4), 150 mM NaCl, 5 mM EDTA, and 1:1,000 Protease Inhibitor Cocktail (#P1860, Sigma-Aldrich). The cell lysates were utilized for co-immunoprecipitation assays using Protein G Dynabeads (#10003D, Invitrogen) as per manufacturer’s instructions. Briefly, Protein G Dynabeads were incubated with 1–10 µg of the specified target antibody, followed by incubation of the bead–antibody complex with the indicated cell lysates. The target antigen was eluted from the bead–antibody–antigen complex using a 50 mM glycine, pH 2.8, elution buffer. The eluates were then used for Western blot analysis.

### Cytometric bead array

Infected mouse BMDM cell supernatant was collected and clarified (centrifugation at 300 × *g* for 10 min, followed by 3,000 × *g* for 10 min at 4°C) to remove cellular debris. The CBA was performed using LEGENDplex Mouse Anti-Virus Response Panel (13-plex) with V-bottom Plate (BioLegend) according to the manufacturer’s instructions. Bead fluorescence was measured with an LSR Fortessa cell analyzer (BD Biosciences) or Cytek’s Aurora System (Cytek), and data were analyzed using the cloud-based LEGENDplex Data Analysis Software Suite (BioLegend).

### Recombinant SARS-CoV-2 viruses

BACmids containing the cDNA genome of mouse-adapted (MA30) SARS-CoV-2 (79) were PCR-amplified into six fragments, each with 30 base-pair overlaps. Fragments were isolated by gel electrophoresis, excised and purified using PureLink PCR reagents, and assembled using Gibson assembly (#E2621L, NEBuilder HiFi DNA Assembly). Assembled products were transformed into NEB 10-beta Competent *Escherichia coli* (#C3019H). All bacterial cultures were grown at 20°C. Chloramphenicol-resistant colonies were selected after 2 days, further amplified in LB24 broth for 3 days, with BACmids then purified using NucleoBond Xtra Midi (TaKaRa Bio) reagents. SARS-CoV-2 recombinant MA30 BACmids with engineered mutations were verified by sequencing. The entire BACmids were fully sequenced. Amounts of each BACmid (1.5 µg) were transfected into 10^6^ Vero-ACE2-TMPRSS2 (Vero-AT) cells using LipoD293 *In Vitro* Transfection Reagent (SignaGen). Cells were then observed for cytopathic effect. Cells were then frozen at −80°C when overt syncytial cytopathologies were evident, typically 3 days post-transfection. Secreted virus titers were determined by plaque development on Vero-AT indicator cells.

### Mouse infections

BALB/c mice (8–10 weeks old) were anesthetized with ketamine-xylazine and inoculated intranasally with 50 µL DMEM solution mixed with the indicated amounts of virus (*n* = 4–5 mice for each virus infection) (79). Weights and survival scores were recorded daily for seven consecutive days. In repeat infections, mice were euthanized at 3 dpi with isoflurane overexposure. Lungs were collected and homogenized in TRIzol (Invitrogen). After centrifuging the samples, total RNA extraction was performed following the manufacturer’s protocol. cDNA was synthesized using M-MuLV reverse transcriptase (New England Biolabs). Then, samples were amplified by RT-qPCR using PowerUp SYBR Green Master Mix (Applied Biosystems). For data analysis, the expression of each gene was averaged from two technical replicates and normalized to *Gapdh* expression using the ΔΔCt method. The qPCR primer sequences are listed in the table below. The qPCR primer sequences for SARS-CoV-2 detection have been reported previously (80). For obtaining lung viral titers, at 3 dpi, mice were euthanized. Lungs were collected and homogenized in PBS. Samples were centrifuged at 1,000 rpm for 5 min. The supernatants were used for infection in plaque assays using VeroE6-TMPRSS2 indicator cells.

### Biosafety

*In vitro* work with infectious SARS-CoV-2 was completed at Loyola University Chicago in restricted access tissue culture rooms under negative air pressure and at BSL2+ containment. *In vivo* SARS-CoV-2 infection studies were completed in BSL2 laboratories at the University of Iowa.

### Statistical analysis

Statistical comparisons were made by the unpaired Student *t*-test, one-way ANOVA, two-way ANOVA, or log-rank test. Error bars indicate the standard error mean of the data. *P* values of less than 0.05 were considered statistically significant.

## Data Availability

The authors confirm that the data supporting the findings of this study are available within the article.
